# Light pollution is greatest within migration passage areas for nocturnally-migrating birds around the world

**DOI:** 10.1038/s41598-018-21577-6

**Published:** 2018-02-19

**Authors:** Sergio A. Cabrera-Cruz, Jaclyn A. Smolinsky, Jeffrey J. Buler

**Affiliations:** Department of Entomology and Wildlife Ecology, University of Delaware, Newark, 19716 USA

## Abstract

Excessive or misdirected artificial light at night (ALAN) produces light pollution that influences several aspects of the biology and ecology of birds, including disruption of circadian rhythms and disorientation during flight. Many migrating birds traverse large expanses of land twice every year at night when ALAN illuminates the sky. Considering the extensive and increasing encroachment of light pollution around the world, we evaluated the association of the annual mean ALAN intensity over land within the geographic ranges of 298 nocturnally migrating bird species with five factors: phase of annual cycle, mean distance between breeding and non-breeding ranges, range size, global hemisphere of range, and IUCN category of conservation concern. Light pollution within geographic ranges was relatively greater during the migration season, for shorter-distance migrants, for species with smaller ranges, and for species in the western hemisphere. Our results suggest that migratory birds may be subject to the effects of light pollution particularly during migration, the most critical stage in their annual cycle. We hope these results will spur further research on how light pollution affects not only migrating birds, but also other highly mobile animals throughout their annual cycle.

## Introduction

Migratory bird species perform seasonal movements between stationary breeding and non-breeding grounds twice every year. While some species migrate during the day (e.g., raptors, aerial insectivores), many others do so at night (e.g., most songbirds, waterfowl and shorebirds). For all of these species, migration may be the most challenging stage of the annual cycle for survival as conditions encountered *en route* are often unfamiliar and unpredictable^1,2^. Nocturnal migrants, in particular, are faced with light pollution, an anthropogenic hazard that has increased rapidly in the last few decades. Light pollution is caused by artificial light at night (ALAN) deployed to illuminate human dwellings but spilling over and spreading into the airspace, reaching areas not inhabited by humans^3^ in the form of skyglow (scatter of light due to particles suspended in the atmosphere). Since the invention of the electrical light-bulb in the 19^th^ century, the use of ALAN and the associated light pollution has increased so dramatically that more than one third of the human population is no longer able to see the Milky Way^4^.

Light pollution has effects on humans and wildlife. Beyond ruining the romantic pastime of stargazing for humans, ALAN has been linked to important physiological and epidemiological maladies such as cancer incidence^5^ and reduced skeletal muscle function^6^. Effects of ALAN on wildlife have been recorded as well^7^. Recent examples include influences on nest site selection by sea turtles^8^, changes in the diversity and behavior of nocturnal moths^9^, and alterations to ecological interactions of insects^10^. Trees in close proximity to sources of artificial lights budburst earlier than trees away from lights^11^. In birds, a positive phototaxis effect (attraction to lights) has been known for a long time^12^. Two of the best documented effects of light pollution on birds is the high mortality due to collision with illuminated buildings and windows^13,14^, and the stranding of seabirds which commonly get drawn by light sources to land^15^. More subtle effects of light pollution on birds are also known, such as disorientation^16^, alterations in reproductive physiology^17,18^, disruption of circadian rhythms^19,20^, and changes of flight behavior^21,22^. Considering the large encroachment of light pollution worldwide^4^ and its known effects on birds, our objective was to test the association of the amount of light pollution (i.e. mean ALAN intensity) within the geographic ranges of nocturnally-migrating birds among five factors including phase of annual cycle, mean distance between breeding and non-breeding ranges, range size, global hemisphere of range, and status of species conservation concern. We were particularly interested in determining if ALAN was relatively greater within the exclusive passage ranges of bird species during migration compared to their distribution ranges during other phases of the annual cycle after controlling for other confounding factors associated with ALAN.

**Figure 1:**
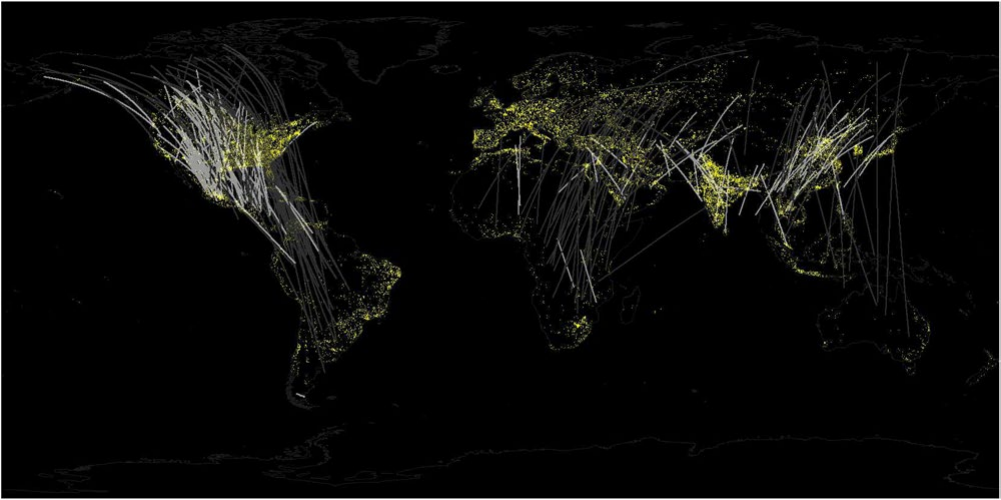
Worldwide distribution of artificial light at night (ALAN - yellow areas over black background). Lines connect centroids of wintering and breeding ranges of nocturnally-migrating bird species considered for analysis (n = 298). Line color represents distances shorter (white) or longer (grey) than the overall median distance. Shorter distances overlap areas with high concentration of ALAN, mainly in North America and eastern Asia. ALAN corresponds to the “vcm-orm-ntl” product (cloud and ephemeral lights free, background set to zero) from the 2015 VIIRS nighttime lights annual composite produced by the Earth Observation Group, NOAA National Geophysical Data Center^62,63^. Map was created with ArcMap version 10.4^66^ (http://desktop.arcgis.com/en/arcmap/).

Despite the widespread use of ALAN, most of the world’s oceans and large water bodies, as well as regions of land at extreme latitudes and within the sub-tropical and tropical latitudes within South America, most of Africa, and most of interior Australia remain largely free of light pollution (Fig. 1). Boreal and tropical regions, where many species of nocturnal migrant birds breed and spend the non-breeding season respectively, have a lower proportion of their land surface permeated by light pollution compared to temperate ecosystems^23^. Hence, we predicted that higher levels of light pollution occur (1) within passage ranges that birds traverse during migration relative to stationary breeding and non-breeding ranges. We assumed that long distance migrants traverse more areas subject to light pollution, and that species with broader distributions incorporate more urban areas, hence we also predicted higher levels of light pollution (2) for species with longer migration distances, and (3) for species with larger geographic ranges. Furthermore, the known adverse effects of light pollution on birds (e.g. mortality due to collision with lit structures, or association with habitat loss from urban development) led us to hypothesize that (4) higher levels of light pollution would be related to species of higher conservation concern. Identifying when during the annual cycle, and whether susceptible species face higher levels of light pollution will aid in directing research efforts to evaluate effects of light pollution in these highly mobile organisms.

## Results

We considered 298 species for analysis, 179 in the western and 119 in the eastern hemisphere (see Methods and Supplementary Table S1). The geographic distribution ranges of nearly all species contained some degree of light pollution during all phases of the annual cycle; the only exception was the migration range of the Golden Crowned Sparrow (*Zonotrichia atricapilla*). The families with the most species used for analysis were Parulidae (Wood warblers, 41 spp.), Anatidae (Waterfowl, 32 spp.), and Scolopacidae (Shorebirds, 32 spp.). The species with the greatest mean light pollution were the Basra reed warbler (*Acrocephalus griseldis*) in its breeding range, and the Sakhalin leaf warbler (*Phylloscopus borealoides*) in both its migration and non-breeding ranges. The full dataset is provided in Supplementary Table S2.

**Figure 2:**
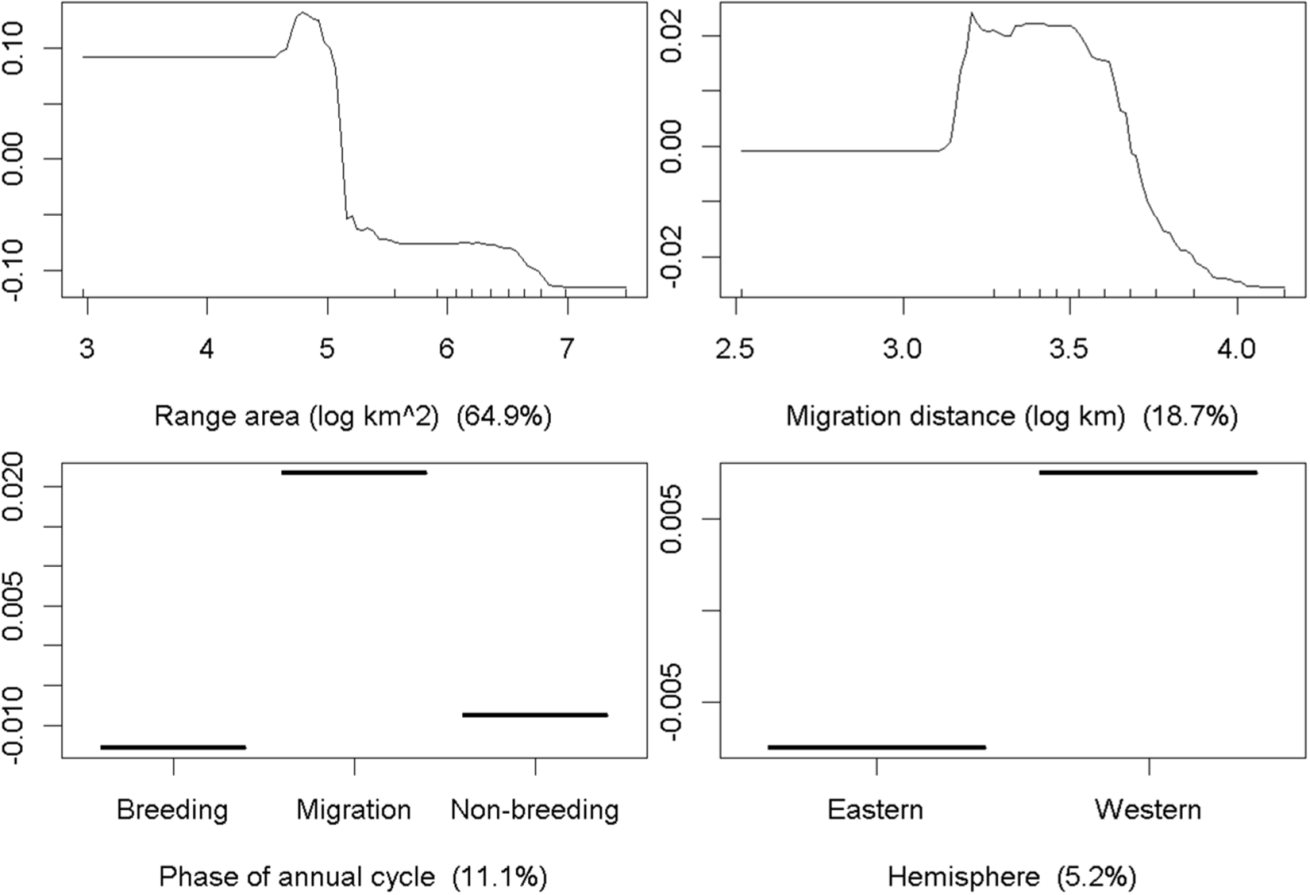
Partial dependence plots of variables associated with intensity of light pollution in the geographic ranges of 298 nocturnally-migrating bird species. Y-axes show the marginal intensity of ALAN in units of 1E9*nanoWatts*cm^−2^*sr^−1^; notice different scales among plots. Rug plots shown inside the top two main plots on the x-axis show deciles of the distribution of predictor values. Values in parenthesis of X-axis label indicate the relative influence of each predictor variable.

**Figure 3:**
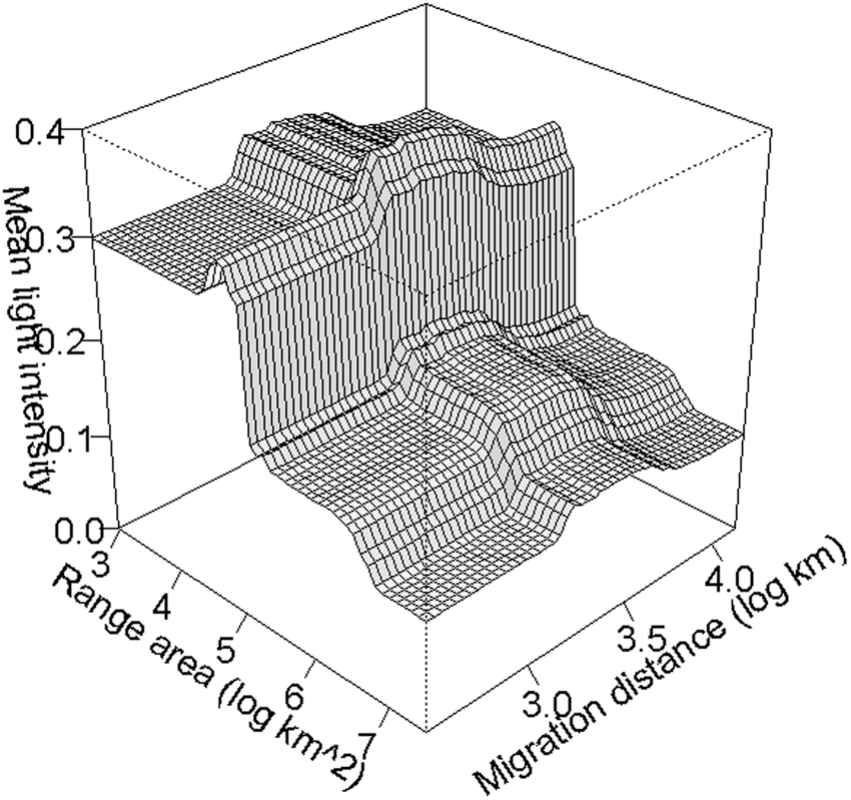
Partial dependence plot for the interactions of seasonal range area and migration distance on the mean artificial lights at night (ALAN) within the geographic ranges of 298 nocturnally-migrating bird species around the world.

The Boosted Regression Tree (BRT) model indicated moderately weak associations among factors with ALAN intensity within geographic ranges (20% of the total deviance explained). Seasonal range size was the most important variable associated with the amount of light pollution, followed by migration distance, phase of annual cycle, and hemisphere (Fig. 2). Consistent with our prediction, we found a higher amount of light pollution in passage ranges compared to stationary ranges. The intensity of light pollution was significantly different between breeding and migration (z = 4.90, n = 897, p < 0.001), and non-breeding and migration ranges (z = −4.16, n = 897, p < 0.001), but not between breeding and non-breeding ranges (z = 0.73, n = 897, p = 0.741) when assessed with Generalized Linear Models. Contrary to our expectations, however, we found lesser mean light pollution for species with longer migration distances and larger seasonal ranges. A strong interaction between range size and migration distance revealed that light pollution varies more widely with migration distance for species with relatively small ranges than for species with relatively large ranges (Fig. 3). Short distance migrants tend to spend their full annual cycle within the bright temperate regions of North America and eastern Asia (Fig. 1) and occupy ranges with higher levels of light pollution than long-distance migrants. We also found that the geographic ranges of species in the Western hemisphere had relatively higher levels of light pollution than those in the Eastern hemisphere. Finally, the association of light pollution with status of conservation concern was extremely weak.

**Figure 4:**
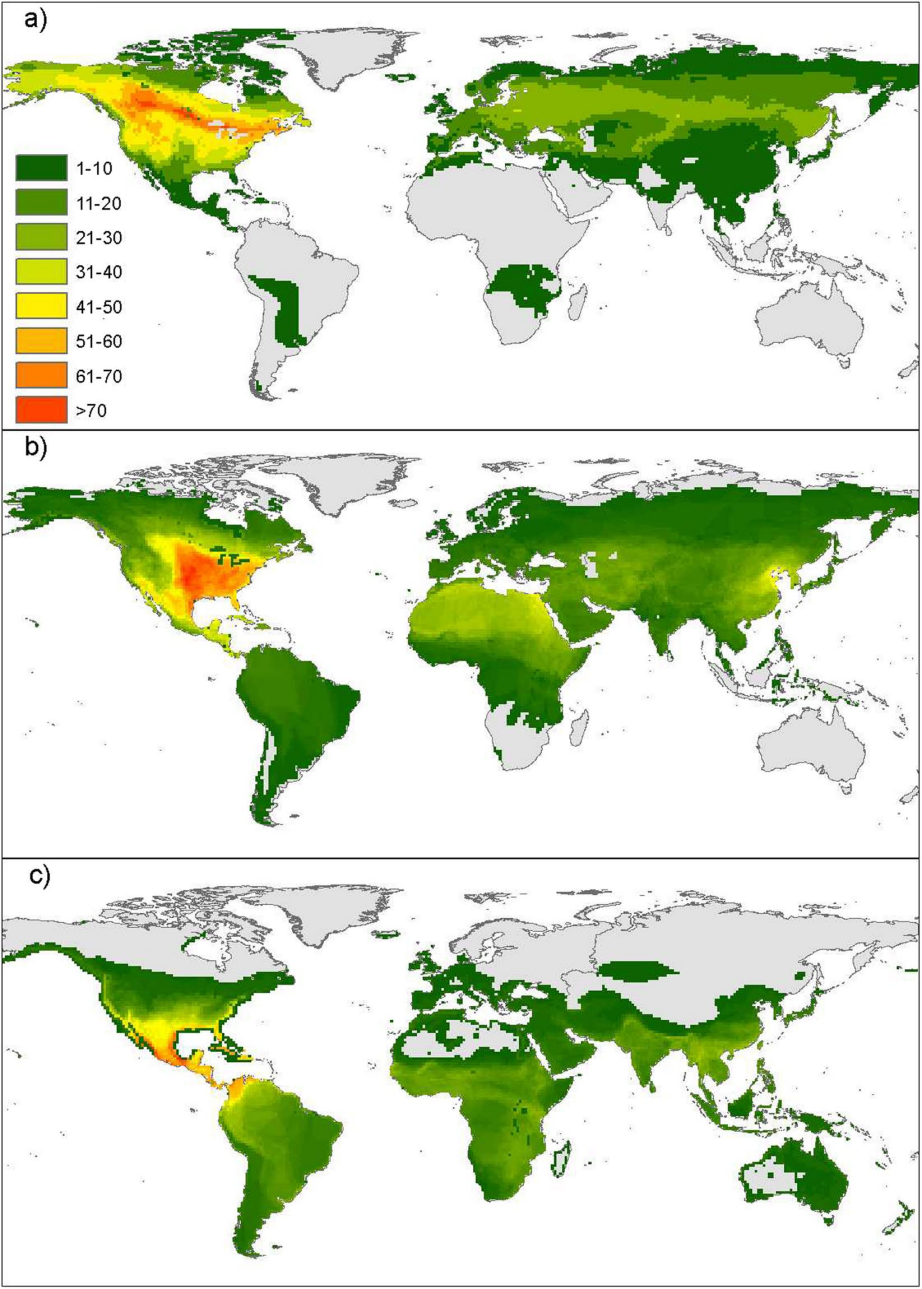
Species richness in (**a**) breeding, (**b**) migration, and (**c**) non-breeding ranges for 298 nocturnally-migrating bird species, estimated by intersecting geographic range map polygons for each phase of annual cycle. This map was created in R version 3.3.2^70^ (https://cran.r-project.org/) with geospatial data from the Birds of the World Geodatabase^64^, and edited with ArcMap version 10.4^66^ (http://desktop.arcgis.com/en/arcmap/).

We inspected the worldwide distribution of the 298 species considered for analysis, and found that the highest species richness occurs in the Western hemisphere (Fig. 4). The highly light-polluted areas in Central and Eastern US hold the highest species richness of migration ranges in the world, while the highest species richness of breeding ranges occur in boreal forests of Canada, and the highest species richness of non-breeding ranges occur in Mexico, along its Pacific and Gulf of Mexico coastlines.

## Discussion

Light pollution was relatively greater within the exclusive passage ranges of nocturnally-migrating bird species during migration compared to their distribution ranges during other phases of the annual cycle after controlling for other factors associated with ALAN. The largest concentrations of ALAN generally occur in urban areas, which occupy about 0.5% of the global land surface, and are mostly concentrated between 30°N and 45°N latitude^24^. Many species of nocturnal migrants breed in boreal forests north of 50° latitude, for example north of the US-Canada border^25^, and winter in tropical areas south of Tropic of Cancer (25°N Latitude). Hence, during migration, long distance migrants traverse latitudes with the highest urban development in the world, leaving from and arriving to areas with low levels of light pollution. Although some land bird species breeding in the Southern Hemisphere migrate to northern latitudes during winter time, bird migration is mainly a Northern hemisphere phenomenon, with most species of migratory land birds moving from North to South between breeding and non-breeding areas respectively^26^. Thus, it appears that many long distance migratory birds are exposed to more extensive ALAN in areas they traverse while aloft during migration, a period of great selective pressure when birds must repeatedly locate stopover habitats where they can safely rest and refuel and contend with weather to fly long distances to reach their destinations in a timely manner. Migration is also the time of year when the ecological impacts of ALAN on the behavior and survival of birds have been well studied^27^. Therefore, we focus our discussion on the impacts of ALAN on birds during migration.

Although ALAN has a small cumulative spatial footprint on the globe, the worldwide distribution of human settlements produces some degree of light pollution within the geographic ranges of all species of nocturnally migrating birds, as it does for mammals^28^ and ecosystems^23^ around the world. Light pollution is present in every continent^4^ except perhaps Antarctica. However, human settlements and artificially-lit-structures are not evenly distributed around the globe^4^. Accordingly, we found the amount of light pollution within migratory bird ranges varied with migration distance, range size, and global hemisphere. These associations were important confounds to control for when assessing the relative association of ALAN among ranges for different phases of the annual cycle. However, they present some interesting patterns in their own right. Short distance migrants may be associated with greater light pollution because they tend to occur within temperate regions where urban development is most widespread. Likewise, since intensity of sky brightness fades with distance from the source^29,30^, species with relatively larger ranges and longer migration distances encompass regions with less human development and have ranges with relatively lower levels of light pollution. Birds migrating through the Nearctic-Neotropical migratory system, in the western hemisphere, move through the US, one of the most urbanized countries in the world^24^, and concentrate during their non-breeding stage in the less urbanized but still light-polluted region formed by Mexico, Central America, and the northern portion of South America^4^. Geographic ranges of bird species in the eastern hemisphere overlap the heavily urbanized and highly light-polluted European Union^4^, but also over Africa and Oceania, the regions in the world with the lowest urbanized fraction^24^, and hence with low encroachment of light pollution. Central and northern Asia, also part of the eastern hemisphere, seem relatively free of light pollution^4^.

At a fine scale, negative consequences have been demonstrated for birds during nocturnal migratory flight. Point sources of ALAN disorient and attract birds actively engaged in migration^27,31–34^ to the extent that birds can be “trapped” by bright sources of ALAN when migrating over urban areas. High-intensity urban light installations can dramatically alter multiple behaviors of nocturnally migrating birds even to distances of several kilometers from the source^35^. Migrants will slow down their flight speed, start circling the light source, and call more frequently near the lights. It is well known that bird mortality due to collisions with buildings is related to light emissions from the buildings^13,14^, but some species appear more susceptible to collision than others^36,37^, suggesting that light pollution has a species-specific effect among nocturnal migrants.

The influence of ALAN on migratory birds also reaches beyond the extent of urban areas. Artificial lights can be perceived from beyond the point sources, particularly during overcast nights^38^. For example, the skyglow of large metropolitan areas may be perceived by an observer at the Earth’s surface from up to 320 kilometers away^39^. Birds aloft will perceive skyglow from even farther away depending on their flight height. Previous research has shown that migrating birds orient towards the skyglow of urban areas, particularly juveniles^40^. Consistent with this behavior, McLaren *et al*.^41^ discovered that total nocturnal migrant bird stopover density on the ground increases monotonically with proximity to areas with bright skyglow over a range of distance up to 200 km. Despite this broad-scale attraction to urban areas, presumably during migratory flight, birds avoid using bright areas at a small scale (1 km radius) for stopping over. Others have found urban sources of ALAN are associated with higher levels of migrant stopover abundance both within green spaces at the interior of urban areas and along urban boundaries^42–44^, supporting the conclusion that observed associations with urban environments during migration^45,46^ are driven, at least in part, by broad-scale attraction to urban sources of ALAN. In contrast, areas containing high levels of ALAN are generally avoided by migratory birds during the breeding and non-breeding seasons^42,46^. Thus, urban sources of ALAN broadly effect migratory behavior and may have a role in shaping migratory routes of individual species, emphasizing the need to better understand the implications of ALAN for migratory bird populations.

Despite the growing body of evidence about the negative effects of light pollution on the environment, the total surface of the planet exposed to light pollution and the brightness of ALAN has increased in recent years^47^. Thus, a call has been made to integrate light pollution in global change research^48^. Continued research on the impact of light pollution on migrating birds at different scales is needed. Future efforts should include continuing to evaluate the influence of ALAN in selection of stopover sites, utilization of green spaces in urban areas, and altering flight behavior. New insights are also needed into the differential effects of ground-based lights as compared to lights in tall structures, and the influence of ALAN in altering the selection of migratory routes, overall migration success, and the onset of migration. For example, light pollution disrupts the timing of bird activities^49–51^, including sleep cycles^19,52^. A combination of methods could provide further insight about the extent and impact of light pollution on migratory birds. Local measures of light pollution could be obtained with light meters^53^ or digital cameras^38^ in stopover sites remotely identified with weather radars^43,54^. Light-level loggers have been used to measure the intensity of light to which birds are exposed in urban and rural environments (reviewed in^55^). This approach could be adapted to provide insight into the levels of ALAN in the stopover sites used by migrating birds. Additionally, increasing day length is related to initiation of developmental stages for spring migration in different bird species (e.g. fattening, hypertrophy of flight muscles, and expression of metabolic enzymes and hormones; reviewed in^56^). Extended periods of activity into the night by birds near urban areas^49^ suggest that some birds may be experiencing increased periods with exposure to light^50^, which has been proven to affect hormone levels and gonadal development^18^. While actual departure for migration depends on factors other than photoperiod^57^, light pollution may alter the onset of migration, particularly for species with small geographic ranges that include urban areas.

## Methods

### Artificial light at night

We mosaicked six geotiff tiles from the Earth Observation Group (EOG) at NOAA National Geophysical Data Center to create a complete dataset of ALAN for the entire world. Each tile contains the mean annual radiance composited from nighttime images taken by the Suomi National Polar-orbiting Partnership (NPP) satellite with its Visible Infrared Imaging Radiometer Suite (VIIRS) Day/Night Band (DNB) during 2015, and are produced in 15 arc-second resolution grids. Although monthly composites are available, we preferred to use annual composites because it prevents the results from being confounded by seasonal variations of snow and vegetation cover^58,59^. The full mosaic spans the globe from 75 N latitude to 65 S. Descriptions of the capabilities of the DNB sensor are available in^60,61^. We used the “vcm-orm-ntl” set of products, which are cloud-free, have had fires (gas flares included) and other ephemeral lights removed, and the background has been set to zero (e.g. moonlight reflection from the earth), leaving just sources of consistent ALAN primarily from electric lighting sources^62^. See^62^ for a full account of the production process of the composited nighttime image used for analysis, and for the complete details of the type of light sources excluded from the dataset. Radiance is measured in units of nanoWatts per square centimeter per steradian (nW cm^2^ sr) but have been multiplied by 1E9^63^. Original radiance values in the individual tiles ranged from −0.12712 to 97386.8. We log_10_-transformed these values to improve its distribution for analysis, adding 1 first to retain all values. The log-transformed values range from −0.059 to 4.988. We converted the polygon geographic ranges of each species (see below) in each phase of annual cycle to raster grids of the same resolution of the ALAN data before calculating the geometric mean of ALAN (i.e. mean of the log_10_-transformed intensity).

### Geographic ranges of birds

We used the Birds of the World geodatabase (BOTW)^64^ to obtain geospatial data characterizing the presence, origin and seasonality of 10,423 bird species around the world. We defined migratory species as those with a distinct “passage” geographic range (“known or thought very likely to occur regularly during a relatively short period(s) of the year on migration between breeding and non-breeding ranges”^65^), and filtered out diurnal migrants. For analyses, we only used data of nocturnal migrants with breeding, migration, and non-breeding ranges identified in the BOTW as extant and native. We cataloged 388 bird species as nocturnal migrants. We examined species with worldwide distribution, and from those with presence in both hemispheres we used data from the hemisphere containing breeding, migration, and non-breeding ranges. We excluded species with migration ranges that sparingly connect breeding and non-breeding ranges (i.e., don’t include most of the land area that migrants fly over during migration). We considered 298 species for analyses. We split the ranges for Nelson’s sparrow (*Ammodramus nelsoni*) into two migratory populations. Thus, we used 299 geographic ranges for each phase of the annual cycle.

### Predictor variables

We classified the geographic ranges of nocturnally migrating species by hemisphere (western: migratory species in the Nearctic-Neotropical system; eastern: all other species). We projected the geographic ranges to the Mollweide equal-area projection in ArcMap 10.4^66^ where we used the “Calculate geometry” tool to estimate the area of seasonal geographic ranges in km^2^. We defined and estimated migration distances as the great circle distance in kilometers between the centroid of each species breeding and non-breeding ranges. We obtained the category of conservation concern for each species from the International Union for Conservation of Nature (IUCN) Red List API^67^. We used the eBird/Clements checklist of birds of the world^68^ to obtain taxonomic Families and current scientific names (scientific names from the BOTW database are not current).

### Analysis

We used the gradient boosting method of Boosted Regression Trees (BRT) to model the variability of mean ALAN for each species of nocturnal migrant (n = 298), with five predictor variables: migration distance (log km), area of geographic range (log km^2^), phase of annual cycle, hemisphere, and IUCN category of conservation concern. BRT does not make assumptions about the data distribution of the response variable, hence it can fit non-linear response functions; in addition, it automatically models interactions among predictors, and can therefore perform better than Generalized Linear Models (GLM) and Generalized Additive Models (GAM)^69^. BRT analysis was performed in R^70^ with the library ‘dismo’ and function ‘gbm.step’, which optimizes the fit of the model through cross validation^71^. We used a tree complexity of 2 to allow two-way interactions among predictors, bag fraction of 0.5, a Gaussian error distribution, and a learning rate of 0.001 to produce a minimum of 1000 trees in the optimized model. The learning rate weighted the contribution of each tree to the model, and the bag fraction specified the proportion of data used to train the model^69^. We compared the mean intensity of light pollution between phases of the annual cycle with a multiple comparison of means (Tukey) post-hoc analysis, after a Generalized Linear Model (GLM) was fitted to our data with the same five predictors as with the BRT. We quantified the worldwide distribution of species richness by overlapping the geographic ranges of the 298 species included in the modeling analyses.

### Data availability

All data generated or analyzed during this study are included in the Supplementary Information files.

## Electronic supplementary material

Supplementary Information
