# Author Correction: Light pollution is greatest within migration passage areas for nocturnally-migrating birds around the world

**DOI:** 10.1038/s41598-018-23089-9

**Published:** 2018-03-14

**Authors:** Sergio A. Cabrera-Cruz, Jaclyn A. Smolinsky, Jeffrey J. Buler

**Affiliations:** Department of Entomology and Wildlife Ecology, University of Delaware, Newark, 19716 USA

Correction to: *Scientific Reports* 10.1038/s41598-018-21577-6, published online 19 Febraury 2018

A supplementary file containing Tables S1 and S2 was omitted from the original version of this Article. This has been corrected in the HTML version of the Article; the PDF version was correct at time of publication.

